# Editorial: Polyamines in plant stress responses and development

**DOI:** 10.3389/fpls.2026.1936743

**Published:** 2026-07-24

**Authors:** Ana Isabel González-Hernández, Loredana Maria Scalschi, Aziz Aziz

**Affiliations:** 1 Superior Polytechnic School of Zamora, University of Salamanca, Zamora, Spain; 2 Department of Biology, Biochemistry and Natural Sciences, University of Jaume I, Castelló, Spain; 3 University of Reims Champagne-Ardenne, INRAE, RIBP UMR 1488, Reims, France

**Keywords:** abiotic stress, crop resilience, endophytes, nano-biotechnology, plant development, polyamines

## 1 Introduction

In the face of accelerating climate change, marked mainly by severe drought events and extreme temperatures, the search for sustainable strategies to enhance crop resilience has intensified. Polyamines (PAs), ubiquitous low-molecular-weight aliphatic nitrogenous bases such as putrescine, spermidine, spermine, and thermospermine, are recognized as critical nitrogenous master regulators of plant growth and cellular development, and pivotal defensive buffers against biotic and abiotic stresses Blázquez, 2024.

This Research Topic brings together outstanding contributions on recent advancements in PA research, from molecular fine-tuning of vascular developmental pathways to innovative nano-biotechnological applications and targeted chemical priming to improve multi-stress resilience. These advances collectively demonstrate how mechanistic insights into PA metabolism, signaling, and delivery can be translated into practical strategies for improving crop performance under increasingly unpredictable environmental conditions.

## 2 Innovations and genetic insights in plant development

At the molecular level, PAs play an indispensable role in normal plant development. Because key PA biosynthetic enzymes are heavily governed by post-transcriptional and translational mechanisms, transcript levels often fail to reflect actual protein abundance. To solve this, Ritchie et al. developed a highly optimized, non-derivatized liquid chromatography–mass spectrometry (LC-MS) toolkit and stable non-radioactive heavy isotope substrates. The authors successfully established a method to simultaneously quantify the kinetic activities of both arginine decarboxylase (ADC) and ornithine decarboxylase (ODC) alongside 11 network-related metabolites in a single MS run. Using this platform to analyze CRISPR-Cas9-engineered tomato, the authors demonstrate that ADC pathway is completely indispensable for floral morphogenesis, since the tomato *adc1 adc2* double mutant presented a complete failure to form flower buds or reproductive organs. ODC pathway remained functional in the *adc1 adc2* plants but could not compensate for the loss of ADC-driven PA synthesis.

A foundational aspect of polyamine biology is its structural and functional specificity. Thermospermine, a structural isomer of spermine, acts as a critical regulator of xylem differentiation. In *Arabidopsis thaliana*, the absence of the thermospermine synthase encoded by *ACAULIS5* (*ACL5*) triggers a severe dwarf phenotype marked by excessive, uncontrolled xylem differentiation. Research presented by Xu et al. demonstrates that *A. thaliana* response to thermospermine is fine-tuned by a delicate homeostatic balance between two basic helix-loop-helix (bHLH) proteins: SUPPRESSOR-OF-ACL51 (SAC51) and LONESOME HIGHWAY (LHW), ensuring proper vascular development. By generating genome-edited upstream open reading frame (uORF) mutants of *SAC51-LIKE2* (*SACL2*) alongside existing family mutants, the authors confirmed that overaccumulation of any single SAC51 family member directly represses LHW function through physical interaction, inducing severe hypersensitivity to thermospermine and restraining excessive xylem proliferation.

## 3 Polyamines in abiotic stress resilience

A primary focus of current research is the use of exogenous PAs to mitigate the detrimental effects of abiotic stresses such as salinity, drought, and flooding. Foliar application of putrescine has been shown to significantly enhance salt tolerance in hybrid poplars (*Populus nigra × maximowiczii*), increasing plant height and stem diameter while alleviating leaf damage and improving metabolic stability (Kundu et al.). The study highlights that putrescine acts as a multifaceted physiological booster: it maintains osmotic balance, improves gas exchange, and stabilizes nitrogen metabolism. By influencing carbon and nitrogen partitioning, exogenous putrescine helps the trees rebuild sucrose pools and sustain growth even in challenging, saline-prone environments. Similarly, the work performed by Wang et al. offers a profound exploration of how exogenous spermine applications can improve drought resistance in alfalfa (*Medicago sativa*). Spermidine application boosts photosynthetic capacity, enhances the antioxidant enzyme system, and the accumulation of signaling substances like abscisic acid.

The challenge of flooding stress, which disrupts root respiration and energy metabolism, is also addressed through PA modulation. Sun et al. provide compelling evidence for the protective role of spermidine in ecological grasses (*Elytrigia elongata)* subjected to flooding stress. Their research highlights how exogenous spermidine acts as a bioactive compound to enhance plant survival under hypoxic conditions. Spermidine application mitigated flooding-induced damage by increasing photosynthetic capacity, regulating antioxidant enzyme activity, and facilitating the accumulation of osmoregulatory substances. These studies collectively provide a theoretical foundation for using PAs as practical agents to improve stress resilience in both woody perennials and ecological grasslands.

Furthermore, technological innovations in PA delivery are expanding the toolkit for precision agriculture. Zhang et al. provided comprehensive insights into how seed priming protects water-saving and drought-resistance in rice. Priming seeds with spermidine or ascorbic acid has been identified as a low-cost, effective technique to promote root growth in rice under drought stress by increasing antioxidative activities and osmoprotectant content, regulating auxin (IAA) biosynthesis and the expression of the *YUCCA* gene family. Moreover, Das et al. present a groundbreaking nano-biotechnological approach to drought tolerance in rice (*Oryza sativa*). By developing putrescine-doped zinc oxide nano-entities (PUT-nZnO), the researchers demonstrated that this formulation effectively facilitates chloroplast survival, restores ion homeostasis, and mitigates drought-induced reactive oxygen species (ROS) and programmed cell death, offering a robust strategy to boost the viability of young seedlings under water deficit conditions.

## 4 Polyamines in plant-microbe interactions

The functional role of PAs further extends to biotic defense mechanisms and beneficial plant-microbe interactions. Augustyniak et al. examined the protective threshold of exogenous PAs in industrial flax (*Linum usitatissimum*) challenged by the soil-borne wilt pathogen *Fusarium oxysporum*. The study revealed that spermidine treatment restricts the development of disease by simultaneously affecting the pathogen’s metabolism and modulating the host plant’s defense responses. These findings highlight the potential of spermidine as a viable candidate for sustainable crop protection strategies. Furthermore, the study of Qin et al. explored the beneficial role of the nitrogen-fixing endophytic bacterium *Klebsiella variicola* DX120E in sugarcane. The application of this endophytic bacterium as a bio-inoculant offers a promising biological approach for modulating plant PA levels. Genomic and transcriptomic analyses revealed that DX120E not only possesses genes essential for PA metabolism, but also stimulates PA metabolism within sugarcane tissues, leading to increased biomass production, alongside up-regulation of key growth-related hormones, such as gibberellins (GAs) and auxins, and increased activity of ROS-scavenging enzymes. Collectively, these findings underscore that leveraging microbe-plant interactions represents a viable and sustainable alternative to conventional chemical fertilizers.

## 5 Conclusion

The integration of physiological, metabolomic, and transcriptomic data presented herein reinforces the status of polyamines (PAs) as master regulators of plant fitness. Whether delivered via foliar or root applications, seed priming, or mediated by beneficial endophytes, PAs offer a versatile framework for optimizing crop performance. Future research must now transition toward long-term field trials to validate the economic and operational feasibility of these PA-based strategies, thereby safeguarding global food and bioenergy security against a backdrop of increasing climate volatility.
